# Shocking

**Published:** 1871-08

**Authors:** 


### SHOCKING

Is that which comes to mind or body with a fearful suddenness and severity; dreadful intelligence causes it to the mind; sudden force to the body; in either case that which is acted upon is the same, it is the nervous system ; whether by bullet or bad news, it is through the nerves that death comes. A man may "nerve" himself up to hear some dreadful information, and may survive it; he may "nerve" himself to meet a shot and survive that; but if either came instantaneously and unexpected, there being no putting forth of the energies of the system to resist, death follows.

A strong, healthy body will survive a shock or stroke which would kill a feeble man instantly. A man of "nerve" will stand up against intelligence which would destroy a weak-minded person. We all instinctively avoid communicating unpleasant intelligence to those in feeble health. It is the suddenness of the shock which makes the consequences terrible; in either case it is the sudden arrest of the flow of the nervous fluid, damming it up as it were in the brain, the seat of life. If a blood-vessel is opened and the mildest liquid in nature is injected against the stream, death is instantaneous; if with the stream, no harm results. All shocks, whether of mind or body, endanger life.

